# Swine Influenza (H3N2) Infection in a Child and Possible Community Transmission, Canada

**DOI:** 10.3201/eid1312.070615

**Published:** 2007-12

**Authors:** Joan L. Robinson, Bonita E. Lee, Jagdish Patel, Nathalie Bastien, Karen Grimsrud, Robert F. Seal, Robin King, Frank Marshall, Yan Li

**Affiliations:** * Public Health and Provincial Laboratory (Microbiology), Edmonton, Alberta, Canada; † University of Alberta, Edmonton, Alberta, Canada; ‡ Alberta Agriculture and Food, Edmonton, Alberta, Canada; § National Medical Laboratory, Winnipeg, Manitoba, Canada; ¶ Alberta Health and Wellness, Edmonton, Alberta, Canada; # Marshall Swine Health Service, Camrose, Alberta, Canada

**Keywords:** swine influenza virus, influenza A virus, H3N2 subtype, human influenza, research

## Abstract

An influenza A virus (H3N2) of probable swine origin, designated A/Canada/1158/2006, was isolated from a 7-month-old hospitalized child who lived on a communal farm in Canada. The child recovered uneventfully. A serosurvey that used a hemagglutination-inhibition assay for A/Canada/1158/2006 was conducted on 54 of the 90 members of the farm. Seropositivity was demonstrated in the index patient, 4 of 7 household members, and 4 of 46 nonhousehold members; none had a history of hospital admission for respiratory illness in the preceding year. Serologic evidence for this strain of swine influenza was also found in 1 of 10 pigs (12 weeks–6 months of age) on the farm. Human infection with swine influenza virus is underrecognized in Canada, and because viral strains could adapt or reassort into a form that results in efficient human-to-human transmission, routine surveillance of swine workers should be considered as part of pandemic influenza preparedness.

## Abstract 2

Seropositivity to the same strain was demonstrated in the child and in multiple other community members.

Influenza A is endemic in a broad range of species, with avian and swine strains having the greatest potential for transmission to humans. Pandemics of influenza A occur when a major change occurs in the proteins of circulating strains of the virus. During the pandemics of the past century, this antigenic shift resulted from reassortment of human and avian strains or adaptation of avian viruses to facilitate person-to-person transmission (*1*). Avian influenza preferentially binds to sialic acid–galactose receptors with an α-2,3 linkage that is abundant on duck intestinal epithelium; human influenza preferentially binds to sialic acid–galactose receptors with an α-2,6 linkage that is abundant on human respiratory epithelium. The respiratory epithelium of swine contains both types of receptors and can potentially be simultaneously infected with avian and human influenza (*2*). Human infection with avian influenza subtype H5N1 is of great concern, with 194 deaths of 321 cases reported worldwide through August 16, 2007 (*3*). Swine infected with avian subtype H5N1 have been identified in Vietnam (*4*), raising the possibility that swine could act as the “mixing vessel” that allows avian influenza (H5N1) to reassort with a human influenza strain, resulting in a virus with high pathogenicity and a high potential for person-to-person spread.

Another theoretical mechanism for the origin of an influenza pandemic would be the adaptation of a swine strain that results in efficient person-to-person transmission, although cross-protection by antibodies to recently circulating human strains may prevent this from occurring with swine influenza virus (SIV) H1 and H3 strains. Infection of humans with SIV was first recognized in 1974 with an H1N1 strain (*5*); the solitary outbreak occurred in military recruits at Fort Dix, New Jersey, USA, in 1976 (*6*). Human infection with SIV subtype H3N2 was first described in Europe in 1993 (*7*). The first reported case of probable infection of a person in North America with a non-H1N1 subtype of SIV occurred in Ontario, Canada, in 2005 with an H3N2 strain detected in the respiratory tract of an adult with no serologic evidence of infection (*8*). We describe a case of SIV (H3N2) infection in a Canadian infant, confirmed by viral isolation and serologic testing.

## Case Report

A 7-month-old boy was admitted to the hospital on September 10, 2006, with a 3-day history of fever, rhinitis, and cough. He had had no previous contact with ill persons. The child was born at term and was hospitalized for 21 days at 5 weeks of age when he received ventilation for 6 days for pneumonia due to respiratory syncytial virus. He lived on a communal farm (90 occupants) with horses, cows, swine, sheep, dogs, cats, turkeys, geese, ducks, and chickens but had no direct contact with the animals. The swine were contained in barns and did not mix with the other animals. His household contacts did not work directly with animals, but his father occasionally spent time in the barns, and his uncle, who lived next door, worked in the swine barns.

On admission, the child was afebrile with a heart rate of 120 beats/min, respiratory rate 56/min, and oxygen saturation of 85% on room air. Diffuse wheeze was noted. Chest radiograph results were unremarkable. Direct fluorescent antibody testing on a nasopharyngeal aspirate was positive for influenza A, and the virus was isolated in rhesus monkey cell culture. The isolate was sent to the National Microbiology Laboratory for influenza subtyping as a requirement of the Canadian influenza surveillance program, where it was subsequently designated A/Canada/1158/2006. The child stayed in the hospital for 2 days and then made an uneventful recovery at home. A cough and rhinitis developed in his 19-month-old brother on the day the index patient was admitted to the hospital, but the brother was not assessed by a physician.

## Methods

### Antigenic Analysis

For the antigenic characterization of A/Canada/1158/2006, hemagglutination-inhibition (HI) assay was performed by using 4 hemagglutination units of virus, 0.7% v/v guinea pig erythrocytes, and postinfection fowl serum specimens for the currently circulating human strains (A/New Caledonia/20/99 [H1N1], A/Wisconsin/67/2005 [H3N2]), past circulating human strains (A/Panama/2007/99 and A/Nanchang/933/95), and swine serum for A/Swine/Texas/4199–2/98 (H3N2) treated with receptor-destroying enzyme (*9*).

### Molecular Characterization

All 8 RNA segments of A/Canada/1158/2006 were amplified by reverse transcriptase–PCR (RT-PCR) and sequenced. A universal primer set for the full-length amplification of all influenza A viruses was used for the RT-PCR (*10*). Viral RNA was extracted from 100 μL of tissue culture fluid with the RNeasy Mini Kit (QIAGEN, Mississauga, Ontario, Canada). Viral RNA was amplified in a OneStep RT-PCR reaction (QIAGEN) following the manufacturer’s recommendations. Briefly, 5 μL RNA was added to the RT-PCR mixture containing 2 μL QIAGEN OneStep RT-PCR enzyme mix, 10 μL 5× QIAGEN OneStep RT-PCR buffer, 400 μmol/L dNTP, 0.6 μmol/L of each primer, and 10 μL Q-solution in a final volume of 50 μL. The conditions used for the Gene Amp 97700 (Applied Biosystems, Streetsville, Ontario, Canada) thermocycler were as follows: 50°C for 30 min for reverse transcription, 95°C for 15 min for the activation of the HotStart DNA polymerase; then 35 cycles of 94°C for 20 s, 58°C for 30 s, 72°C for 4 min, followed by an extension of 10 min at 72°C. The PCR products were purified by using QIAquick PCR purification kit (QIAGEN) and sequenced on an ABI 377 Sequencer, using a fluorescent dye-terminator kit (Applied Biosystems). The DNA sequences were assembled and analyzed with SEQMAN, EDITSEQ, and MEGALIGN programs in Lasergene (DNASTAR, Madison, WI, USA). Phylogenetic trees were generated by the neighbor-joining method using the MEGA program (*11*).

### Serologic Testing

Once it became evident that A/Canada/1158/2006 was closely related to swine influenza viruses, HI was performed on serum specimens collected from the index patient, the symptomatic sibling, and both parents 29 days after the hospitalization. To further investigate the spread of SIV to humans, approval was then granted by the Health Research Ethics Board of the University of Alberta to obtain information and serum specimens from other members of the communal farm. The study team visited the farm 3 months after the hospitalization of the index patient and explained the study to the occupants. Serum specimens were then collected from the other 4 siblings of the index patient and 46 other occupants who lived in a total of 17 households. Participants provided the following data: age, exposure to swine (none, <1 hour/week, or >1 hour/week), and history of influenza-like illnesses (ILI; defined as cough and fever) in the preceding year. Serum samples were tested by using an HI assay against the currently circulating human strains A/New Caledonia/20/99 (H1N1), A/Wisconsin/67/2005 (H3N2), and the isolate from the index patient, A/Canada/1158/2006. HI titers were defined as the reciprocal of the highest dilution of serum that completely inhibited hemagglutination of a 0.7% solution of guinea pig erythrocytes. Specimens were considered seropositive for influenza virus at a titer of >32.

### Swine Investigation

The purpose of these investigations was to determine the extent of recent swine influenza in swine on the farm and to look for evidence of infection with the SIV strain isolated from the index child. The history of influenza or unexpected respiratory illness in the swine on the farm was obtained. Nasal swabs were obtained from grower pigs (4 to 16 weeks of age) and processed by RT-PCR for influenza A matrix gene. Serologic testing for influenza, using an ELISA for H1N1 and H3N2 strains and HI for A/Canada/1158/2006, was performed on samples from grower-finisher pigs (12 weeks to 6 months of age). Five grower pigs that were doing poorly were killed and pulmonary autopsies were performed. All swine used in these investigations were on the farm at the time the index child was ill.

## Results

### Antigenic and Molecular Characterization of A/Canada/1158/2006

Initial HI testing showed that the isolate was not inhibited by antiserum against recent (A/Wisconsin/77/2005 and A/New Caledonia/20/99) and past (A/Panama/2007/99 and A/Nanchang/933/95) human influenza A strains but was inhibited by antiserum against A/swine/Texas/4199–2/98 (H3N2) virus with HI titer of 128. These findings indicate that the A/Canada/1158/2006 virus was antigenically related to SIV (Table 1). The results also indicate that the assay is specific because no cross-reactivity was observed between the human reference strain antiserum and the swine influenza viruses (Table 1). Nucleotide sequences of the full-length coding regions of all 8 RNA segments of the isolate further determined that it was most closely related to A/swine/Ontario/33853/2005 (H3N2) virus, which shares the same human/classic swine/avian triple reassortant genotype as the H3N2 subtype viruses that emerged in swine in the United States in 1998 (*8*). Sequence analysis showed that nucleic acid homology between A/Canada/1158/2006 and A/swine/Ontario/33853/2005 ranges from 98.4% (HA) to 100% (M1), and that amino acid (aa) identities range from 97.9% (HA) to 100% (NP, NS2, M1). A deletion of 4 aa at position 156–159 was observed in the HA1 region of the A/Canada/1158/2006 HA protein. Amino acid substitutions were found in the HA (HA1 domain: G7, K142, S162; HA2 domain: T77, Q139, M149, E150, N160), neuraminadase (NA) protein (P45, K74, N150, M349, L354), NS1 (M112), PB1 (K211, D738), PB2 (K368, S661, T722), and PA (V44, R99, I42) proteins. Phylogenetic analysis showed that all of the genes of A/Canada/1158/2006 clustered with Canadian swine isolates from 2005 (*9*) (data not shown). Nucleic acid identity between the HA and NA genes of A/Canada/1158/2006 and the current vaccine strain A/Wisconsin/67/2005 was 90.9% and 94.6%, and the aa identities were 90.2% and 94.5%, respectively.

**Table 1:** Hemagglutination-inhibition reaction of A/Canada/1158/2006 isolates with reference antiserum against currently circulating human and swine viruses

Antigen	Antiserum (titers)				
	A/New Caledonia/ 20/99 (human H1N1)	A/Wisconsin/ 67/2005 (human H3N2)	A/Panama/ 2007/99 (human H3N2)	A/Nanchang/ 933/95 (human H3N2)	A/Swine/Texas/ 4199–2/98 (swine H3N2)
Control					
A/New Caledonia/20/99 (human H1N1)	320	<4	<4	<4	<4
A/Wisconsin/67/2005 (human H3N2)	<4	320	64	8	8
A/Ontario/RV1273/2005 (swine H3N2)	<4	<4	<4	<4	256
Patient					
A/Canada/1158/2006	<4	<4	<4	<4	128

### Serologic Testing

Seropositivity (HI titer >32) to A/Canada/1158/2006 was demonstrated in the index patient, the symptomatic sibling, 1 asymptomatic sibling, and both parents (Table 2, household A). Three other siblings were seronegative. Four children from 2 other households were also seropositive (Table 2, households B and C); the father from household B, 1 other child from household B, and the mother from household C were seronegative. The father from household C worked in the swine barn but was unavailable for testing. History of ILI within the preceding 12 months in seropositive participants was reported only for the index patient and for a 3-year-old girl from household C who was not hospitalized or tested for influenza virus during her illness. Seronegative results were obtained from another 20 adults (14 women and 6 men) and 19 children (8 girls and 11 boys) from 14 different households. For these households, swine exposure was reported as none for 9 adults and 7 children, <1 hour/week for 11 adults and 8 children, and >1 hour/week for 4 children including 3 teenagers who worked in the swine barns. When serum samples from the 54 participants in the study were tested for HA-specific antibodies to the current human influenza A virus H3N2 and H1N1 subtypes, one of the patients who was seropositive for SIV at a titer of 32 had an identical titer for A/Wisconsin/67/2005 (H3N2) (Table 2), and one of the adults who was seronegative for SIV had a titer of 32 for A/New Caledonia/20/99 (H1N1) (data not shown). All other persons tested were seronegative for the 2 human strains of influenza.

**Table 2:** Clinical features and hemagglutination-inhibition reaction of positive antiserum from 9 members of 3 different households of a communal farm with recently circulating swine influenza (H3N2) virus A/Canada/1158/2006*

Household	Age, y	Sex	A/Wisconsin/ 67/2005 titer	A/New Caledonia/ 20/99 titer	A/Canada/ 1158/2006 titer	Swine exposure	Clinical features
A (index patient)	0.6	M	<4	<4	256	None	Hospitalization with ILI and isolation of swine influenza
A†	1	M	<4	<4	256	None	None (URI coincident with ILI in index case)
A	35	F	<4	<4	32	None	None
A	38	M	8	<4	32	<1 h/wk	None
A	8	M	<4	<4	64	<1 h/week	None
B	7	M	32	<4	32	<1 h/wk	None
C	8	M	4	<4	64	>1 h/week	None
C	5	M	<4	<4	128	<1 h/week	none
C	3	F	<4	<4	128	None	ILI 1 mo before index case

*URI, upper respiratory illness; ILI, influenza-like illness. †Symptomatic sibling.

### Swine Investigation

Influenza (H3N2) was last documented in the swine herd in September 2005. The herd received breeding animals from a Manitoba herd, where swine influenza of an unknown subtype had recently been documented. Nasal swabs collected from 25 grower pigs ≈3 weeks after the index child was ill were negative for SIV. Serum specimens obtained from 10 grower-finisher pigs were all negative by ELISA for swine influenza (H1N1), but 4 were positive for swine influenza (H3N2) strains, with 1 of these 4 strains being seropositive for A/Canada/1158/2006 by HI assay (HI titer 32). Results of the lung autopsies all showed evidence of subacute bronchointerstitial pneumonia, varying from mild to moderate. Lesions typical for swine influenza were not noted, but an initial insult due to SIV could not be excluded.

## Discussion

We describe an infant with virologic and serologic evidence of infection with SIV (H3N2) and an ILI. Serologic evidence of infection with the same strain was found in 4 of 7 household members and in 3 of 46 nonhousehold contacts, with only 1 of the seropositive patients having a history of an ILI within the preceding year, which demonstrated unrecognized human infection with SIV. This relatively high seroprevalence is in contrast to a recent outbreak of avian influenza (H7N3) in which seropositivity was not documented in 91 persons exposed to infected poultry, including 2 poultry workers from whom the virus was isolated (*12*). The difference in the apparent incidence of infection may be explained in part by the fact that culling of infected poultry occurred immediately; in our study, infection of swine was not recognized and long-term human exposure may have occurred.

Infection of swine with human influenza viruses has been recognized for decades (*2*); in a recent US study, 22.8% of pigs were seropositive for human influenza viruses, although some may have had vaccine-induced immunity (*13*). Swine influenza (H3N2) emerged in 1998 in the United States, where subtype H1N1 viruses had predominated for 60 years (*2*). The isolate from this current study is closely related to triple reassorting genotype viruses that spread rapidly throughout the US swine population and have HA, NA, and RNA polymerase (PB1) genes of human influenza virus lineage; nucleoprotein, matrix, and nonstructural genes of classic swine influenza (H1N1) lineage; and RNA polymerase (PA and PB2) genes of North American avian virus lineage (*8*). However, triple reassortant SIV was not documented in swine in Canada until 2005 (*8*), which makes it unlikely that human cases occurred before that year and that seroreversion had occurred in any of the persons in the current serosurvey.

A previous study showed cross-reactivity in HI assay between the vaccine strain A/Panama/2007/99 reference antiserum and the triple reassortant A/swine/Minnesota/593/99, which is not unexpected since the HA gene of the triple reassortant viruses is a descendant of human viruses that circulated in 1995 (*14*,*15*). However, no cross-reactivity was observed between the reference human strain antiserum and the isolate from this study, which suggests that the seroconversion observed was indeed due to infection with swine influenza (H3N2) and not to cross-reactive antibody to human influenza (H3N2) infection. The low rate of seropositivity to recently circulating strains of human influenza in the study is likely explained by the fact that the farm is a relatively closed community. The child who was seropositive for both human and swine influenza viruses was likely exposed to both viruses. The HA protein of A/Canada/1158/2006 diverges significantly from the one of A/Wisconsin/67/2005, and antiserum against A/Wisconsin/67/2005 does not inhibit A/Canada/1158/2006 in HI assay.

Swine influenza (H3N2) has recently reassorted with H1N1 strains to produce H1N2 subtypes and has spread to turkeys in the United States (*16*) and Canada (*8*). A 4-aa deletion was found in the HA protein of A/Canada/1158/2006 when compared with similar swine influenza (H3N2) strains currently circulating in North America. This region of the protein has been assigned to antigenic sites (*17*) and has been associated with adaptation to growth in eggs (*18*). Phylogenetic analysis showed that each of the 8 viral genes of A/Canada/1158/2006 clustered with A/swine/Ontario/33853/2005 (H3N2) and other swine/turkey Canadian isolates from 2005. Although the HA gene of these isolates were shown to be closely related to American viruses that were first isolated from pigs in 1999, they represent a new distinct cluster (*2*). The NA genes are phylogenetically distinct from the US swine isolates and are represented by human influenza (H3N2) isolates from Asuncion, Paraguay (2001), and New York (2003) (*2*).

A recent review described 50 cases of symptomatic human infection with SIV, documented in the literature through April 2006; 46 cases were infected with subtype H1N1 and 4 were infected with subtype H3N2 (*19*). The spectrum of pathogenicity of SIV infection ranges from asymptomatic infection (*6*) to death; 7 of these 50 patients died (*5*,*20*–*24*). Laboratory-confirmed swine influenza in humans may be “the tip of the iceberg.” Diagnosis of the current case was serendipitous because typing was performed only because the case occurred outside of influenza season.

The mode of spread of SIV in humans is not established. Because of his young age, the index patient was not likely to have had unrecognized direct contact with swine. That aerosolization of influenza virus occurs is increasingly recognized (*25*), but the child was reportedly never in the barns that housed the swine. However, other members of the farm reported that infants were sometimes taken for walks through the barn. The child also may have acquired the virus from person-to-person spread or from fomites. All 13 patients in the Fort Dix outbreak and 15 of 37 previously reported civilian case-patients also had no swine contact (*19,**20*).

The Fort Dix outbreak of SIV in humans lasted only 21 days and never spread outside the military base. The calculated basic reproductive rate (R_0_) was only 1.1 to 1.2. This suggests that person-to-person spread of the implicated H1N1 strain was not efficient enough to produce a major epidemic (*26*). However, future strains of SIV could have a higher R_0_, and documentation of a case of swine influenza (H3N2) in a child with unrecognized transmission within the community adds another possible mechanism by which major epidemics of influenza could arise. Swine influenza infection in humans most commonly results in either no symptoms or a self-limited illness (*6*). However, routine surveillance for cases among swine workers may enable early detection of a strain with the potential for person-to-person transmission, prompting institution of infection control measures and vaccine development.
